# Pediatric Chronic Monteggia Fractures: Insights From a Comprehensive Review

**DOI:** 10.1002/pdi3.70033

**Published:** 2025-12-23

**Authors:** Gengze Li, Yuan Zhang

**Affiliations:** ^1^ Department of Orthopedic Surgery Center for Joint Surgery The Second Affiliated Hospital of Chongqing Medical University Chongqing China; ^2^ Department of Orthopaedics Children's Hospital of Chongqing Medical University National Clinical Research Center for Child Health and Disorders Ministry of Education Key Laboratory of Child Development and Disorders Chongqing Key Laboratory of Structural Birth Defect and Reconstruction Chongqing China

**Keywords:** chronic Monteggia fracture, diagnosis, pediatric, radial head dislocation, treatment

## Abstract

Monteggia fractures represent relatively infrequent injuries in the pediatric population, accounting for approximately 2% of all forearm fractures. However, the rate of missed diagnoses ranges from 30% to 50%, leading to the development of chronic Monteggia fractures in children. Chronic Monteggia fractures frequently result in elbow deformities and functional limitations, significantly impacting the patients' quality of life. The classification systems and treatment approaches for this condition are complex, and therapeutic strategies remain a subject of considerable debate. This review comprehensively examines the definition, incidence, etiology, classification, pathophysiological characteristics, diagnosis, treatment strategies, and prognosis of chronic Monteggia fractures in children. The aim of this study is to provide a reference for further research into the management of this challenging pediatric orthopedic condition.

## 1 Introduction

Chronic Monteggia fracture in children is a rare but challenging disease in pediatric orthopedics. Despite Monteggia fracture accounting for less than 2% of all forearm fractures in children [1, 2, 3], this condition has a high misdiagnosis rate, ranging from 30% to 50% for various reasons, such as atypical imaging findings, a lack of unified and complete classification, and oversight in clinical and imaging assessments [1, 2, 4, 5, 6]. Patients often present with elbow dysfunction, deformity, pain, and potential nerve injuries [1, 4, 7, 8], significantly impacting their quality of life. Furthermore, the management of chronic Monteggia fractures is significantly more complex than that of acute cases. Although closed reduction of the dislocated radial head and realignment of the ulna, followed by internal fixation via K‐wires or elastic stable intramedullary nailing (ESIN), typically yields favorable outcomes in acute cases [9], chronic cases often require more intricate surgical procedures. These methods may include ulnar osteotomy, open reduction of the radial head, and reconstruction of the annular ligament [8, 10, 11]. Moreover, certain aspects of treatment remain controversial, particularly the necessity of annular ligament reconstruction [10, 12], which continues to challenge consensus and may impact treatment efficacy.

Therefore, it is particularly crucial to review the definition, incidence, causes of misdiagnosis, classification, pathophysiology, diagnosis, treatment strategies, and prognosis of chronic Monteggia fractures.

## 2 Definition

Chronic Monteggia fracture in children refers to a fracture of the proximal third of the ulna shaft combined with dislocation of the radial head, where the injury has not been treated in a timely manner or the initial treatment has failed for more than 4 weeks [1, 6, 7, 13, 14]. Monteggia equivalent fractures have a similar injury mechanism to Monteggia fractures but do not lead to separation of the proximal radioulnar joint and may manifest as a fracture of any part of the ulna combined with a fracture of the radial neck or other complex elbow joint injuries [6, 15]. Patients with chronic Monteggia fractures often develop malunion or chronic radial head dislocation due to a missed diagnosis or poor reduction [6, 16] and often present with elbow dysfunction, elbow deformity and pain, which seriously affects the patient's daily life.

## 3 Incidence Rate

The incidence of chronic Monteggia fractures in children lacks definitive epidemiological data, and its occurrence is closely associated with the rate of missed diagnoses during initial presentation. According to relevant studies, Monteggia fractures in children are relatively rare, accounting for 0.4%–1% of all forearm fractures [1, 2, 3], with 30%–50% of cases potentially missed during the initial visit [1, 2, 4, 5, 6]. Research studies indicate that ulnar fractures are readily apparent, whereas radial head dislocations are prone to missed diagnosis, particularly when associated with plastic deformation or greenstick fractures of the ulna [1, 17]. This oversight can subsequently lead to the development of chronic Monteggia fractures.

## 4 The Etiology of Chronic Monteggia Fractures in the Pediatric Population

### 4.1 Atypical Radiographic Presentation

In pediatric patients, incomplete ossification of the radial head epiphysis can lead to an atypical presentation of radial head dislocation or subluxation on radiographs, potentially mimicking normal epiphyseal imaging [6, 18]. The unossified secondary ossification centers may appear normal on X‐rays, whereas the “empty zone” composed of abundant cartilage can obscure the injury, leading to missed diagnoses [18]. Patients with some Monteggia fracture variants exhibit only plastic deformation of the ulna without discernible fracture, making identification challenging via X‐ray and potentially misdiagnosed as other conditions, such as coronoid fracture [19, 20]. Furthermore, the reliability of the radiocapitellar line (RCL) in infants and young children is limited, potentially due to age, sex, and anatomical variations, which can interfere with accurate diagnosis [21]. Research indicates that radial shaft bowing at the tuberosity can complicate the application of the radiocapitellar line [19]. Consequently, conventional X‐ray examinations have limited sensitivity in some patients, often necessitating ultrasound [18] or arthrography for diagnostic support. However, these advanced imaging techniques are not yet universally available in emergency settings, increasing the risk of missed Monteggia fracture diagnoses.

### 4.2 Omissions in Clinical Examination and Radiological Assessment

A full‐length radiographic assessment of the forearm, including the elbow joint, is essential for the accurate diagnosis of pediatric Monteggia fractures. Singh et al. [22] emphasized that neglecting to evaluate the elbow joint radiographically—while focusing solely on ulnar fractures—is a major contributor to missed diagnoses in children with Monteggia fractures. In patients presenting with forearm or elbow injuries, careful assessment of radiocapitellar alignment and ulnar integrity is imperative. Clinically, evaluations should focus on the alignment of the radiocapitellar joint. Palpation of the radial head can assist in determining the presence of dislocation.

### 4.3 Concomitant Injuries and Inexperienced Clinicians

The inaccurate interpretation of pediatric elbow radiographs by emergency physicians without an orthopedic background, as well as by junior orthopedic clinicians with limited experience, coupled with excessive reliance on radiology reports and a lack of familiarity with Monteggia‐equivalent lesions, significantly increases the likelihood of missed diagnoses of pediatric Monteggia fractures [6, 22, 23]. Furthermore, the presence of concurrent injuries in certain patients may divert the clinician's attention, leading to further diagnostic oversights.

## 5 Classification

Current classification systems for Monteggia fractures, such as the Bado classification, are designed primarily to categorize acute fractures on the basis of the direction of radial head dislocation and associated ulnar fractures. However, these systems do not fully address the complexities of chronic Monteggia fractures, which often arise from delayed diagnosis or inadequate management during the acute phase. Despite being tailored for acute injuries, understanding these classifications is crucial for managing chronic cases, as they help identify the long‐term consequences of the injury, guide treatment decisions, and differentiate chronic Monteggia fractures from other pathologies. The acute fracture type directly influences chronic presentation; thus, a thorough understanding of these classifications is essential for developing appropriate strategies for managing chronic Monteggia fractures and improving patient outcomes.

### 5.1 Bado Classification and Jupiter Subtype Classification

**FIGURE 1:**
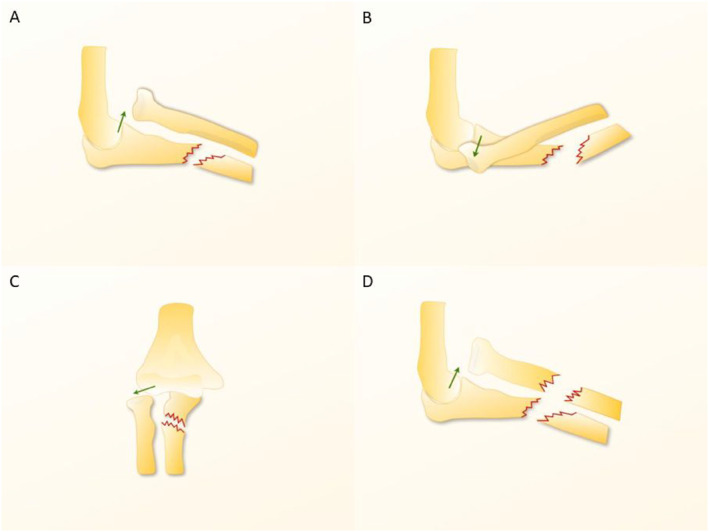
Bado classification for Monteggia fracture locations. (A) Bado type I: anterior radial head dislocation and fracture of the proximal or middle third of the ulna. (B) Bado type II: posterior radial head dislocation and fracture of the proximal or middle third of the ulna. (C) Bado type III: lateral radial head dislocation with a fracture of the ulnar metaphysis. (D) Bado type IV: radial head dislocation in any direction with a fracture of the proximal or middle third of the ulna and radius. Reproduced from Ref. 31 with permission from ELSEVIER, copyright 2014.

The Bado classification (Figure 1), introduced by Jose Luis Bado in 1967, categorizes Monteggia fractures on the basis of the direction of radial head dislocation [6, 24, 25, 26]. Specifically, Bado type I fractures, characterized by anterior radial head dislocation and fracture of the proximal or middle third of the ulna, represent the most frequently encountered variant. The mechanisms of injury in this subtype include (1) an indirect mechanism involving hyperpronation, leading to radial head dislocation with subsequent load transfer to the ulna, resulting in fracture; (2) an indirect mechanism involving hyperextension, inducing reflex contraction of the biceps muscle, causing radial head dislocation followed by ulnar fracture; and (3) a direct mechanism, where a posterior ulnar impact results in a posteriorly angulated ulnar fracture, subsequently dislocating the radius (nightstick injury) [6, 26]. Bado type II fractures, involving posterior radial head dislocation and fractures of the proximal or middle third of the ulna, are less common. The presumed mechanism involves an indirect, longitudinal force applied to a partially flexed elbow, resulting in a posterior ulnar cortex fracture [6, 26]. Bado type III, which presents as lateral radial head dislocation with a fracture of the ulnar metaphysis, is relatively common, typically resulting from forced varus stress applied to an extended elbow, often accompanied by posterior interosseous nerve injury [6, 26]. Bado type IV fractures, characterized by radial head dislocation in any direction with a fracture of the proximal or middle third of the ulna and radius, are exceedingly rare, with unclear injury mechanisms, and are more frequently observed in adults [6, 26].

Given the immature skeletal development in pediatric patients, characterized by increased bone elasticity, joint flexibility, and ligamentous laxity, certain individuals, when subjected to the same injury mechanisms as Monteggia fractures, may not exhibit proximal radioulnar joint dissociation, and their radiographic presentation and therapeutic approaches mirror those of Monteggia fractures [15, 25]. Jose Luis Bado termed these presentations Monteggia equivalent fractures, which primarily encompass Bado types I and II [15, 25]. Bado type I equivalents include (Ia) anterior dislocation of the radial head; (Ib) fracture of the ulnar diaphysis with a fracture of the neck of the radius; (Ic) fracture of the neck of the radius; (Id) fracture of the ulnar diaphysis with a fracture of the proximal third of the radius; and (Ie) fracture of the ulnar diaphysis with an anterior dislocation of the radial head and a fracture of the olecranon [15, 25]. The equivalent of Bado type II dislocation is posterior radiocapitellar joint dislocation associated with epiphysis or radial neck fracture [15, 25]. With advancing research, Ravessoud et al. [27] classified lateral condylar fractures and ipsilateral ulnar shaft fractures as Bado type III equivalents [28]. Furthermore, Arazi et al. [29] categorized an ipsilateral supracondylar humerus fracture and a distal radius fracture as a Bado type IV equivalent [28], thereby expanding the understanding of this domain.

**FIGURE 2:**
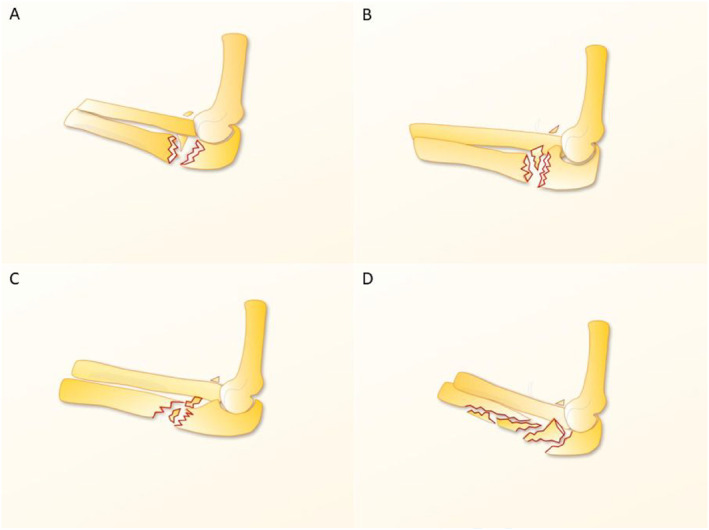
Jupiter classification for Bado II Monteggia fracture dislocations. (A) Type IIA, ulnar fracture involving the olecranon and coronoid processes; (B) type IIB, ulnar fracture at the metaphyseal–diaphyseal junction distal to the coronoid process; (C) type IIC, ulnar fracture at the diaphyseal level; and (D) type IID, comminuted fracture extending from the olecranon to the diaphysis. Reproduced from Ref. 31 with permission from ELSEVIER, copyright 2014.

The Bado classification system offers a concise framework for the rapid identification of common Monteggia fracture patterns [30, 31], thus facilitating clinical decision‐making in diagnosis and treatment. However, the Bado classification is limited to four frequently observed Monteggia fracture types, and it does not encompass injury patterns involving medial or anteromedial radial head dislocation. Consequently, Segaren et al. [30] expanded the Bado classification by designating medial or anteromedial radial head dislocation with proximal ulna fracture as Bado type V. Furthermore, the Bado classification does not incorporate injuries to critical anatomical structures such as the olecranon and coronoid processes, thereby failing to fully represent the severity of the fracture. Jupiter et al. [32] proposed a subclassification of Bado type II fractures on the basis of the location and type of ulnar fracture and the pattern of radial head injury (Figure 2). This subclassification includes the following: type IIA, ulnar fracture involving the olecranon and coronoid processes; type IIB, ulnar fracture at the metaphyseal–diaphyseal junction, distal to the coronoid process; type IIC, ulnar fracture at the diaphyseal level; and type IID, comminuted fracture extending from the olecranon to the diaphysis [31, 33].

### 5.2 Letts Classification

**FIGURE 3:**
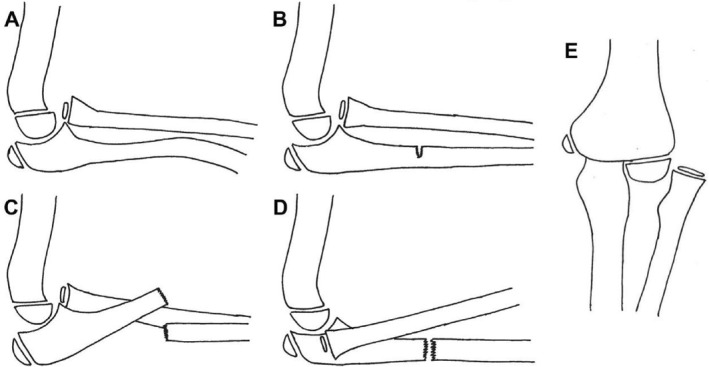
Letts classification for Monteggia fracture dislocations. (A) Letts Type A, which is characterized by anterior bend of the ulnar shaft accompanied by anterior radial head dislocation (plastic deformation); (B) Letts Type B, which involves a greenstick fracture of the ulnar shaft with anterior radial head dislocation; (C) Letts Type C, which is defined by a complete fracture of the ulnar shaft with anterior radial head dislocation; (D) Letts Type D, which presents with an ulnar shaft fracture and posterior radial head dislocation; and (E) Letts Type E, which involves an ulnar shaft fracture with lateral radial head dislocation. Reproduced from Ref. 7 with permission from ELSEVIER, copyright 2020.

The Letts classification (Figure 3), as proposed by Letts et al. in 1985 [34], provides a more detailed system for classifying Monteggia fractures, building upon the Bado classification. It is particularly well suited for the pediatric population, as it incorporates equivalent fracture patterns such as plastic deformation and greenstick fractures. Consequently, the Letts classification provides greater clinical utility than the Bado system [14, 31].

The Letts classification system categorizes pediatric Monteggia fractures into five distinct types, differentiated by the morphology of the ulnar fracture and the direction of radial head dislocation: Letts Type A, which is characterized by anterior bend of the ulnar shaft accompanied by anterior radial head dislocation (plastic deformation); Letts Type B, which involves a greenstick fracture of the ulnar shaft with anterior radial head dislocation; Letts Type C, which is defined by a complete fracture of the ulnar shaft with anterior radial head dislocation; Letts Type D, which presents with an ulnar shaft fracture and posterior radial head dislocation; and Letts Type E, which involves an ulnar shaft fracture with lateral radial head dislocation [31, 34].

### 5.3 Alternative Classification Systems

Olney et al. [35] and Čepelík et al. [36] classified Monteggia fractures on the basis of the radial head's condition and clinical experience into three categories. Group I, (true) Monteggia lesions, are characterized by an ulnar fracture at any level with concurrent proximal radius dislocation. The primary treatment objective is to maintain ulnar length and stability while reducing the radial head. Group II, displaced Monteggia equivalent, is defined by an ulnar fracture at any level with a displaced proximal radius fracture in any direction, with a mean displacement (> 1° according to Judet's classification). The key to treatment is the restoration of the anatomical proportions of the proximal radius. Group III, nondisplaced Monteggia equivalent, is defined by an ulnar fracture at any level with a nondisplaced proximal radius fracture (≤ 1° according to Judet's classification). This variant often allows for nonoperative management with a favorable prognosis.

In 1985, Wiley and Galey emphasized the impact of olecranon and proximal ulnar fractures on the radiocapitellar joint, dividing Monteggia fractures into three types. Wiley's I–III described anterior, posterior, and lateral radiocapitellar joint dislocation, each associated with an olecranon fracture [28, 37]. This classification system was the first to incorporate olecranon injuries within the scope of Monteggia fracture equivalents, offering novel perspectives for clinical diagnosis and treatment.

Given the complexity and diversity of Monteggia fracture equivalents and the limitations of existing classification systems, Xu et al. [28], through a comprehensive analysis and review of the literature, proposed a new classification system for Monteggia fracture equivalents, defined as an ulnar fracture at any level combined with a proximal radial fracture. Building upon the classifications of Olney and Čepelík et al., this system focuses on the status of the radiocapitellar joint and divides such injuries into three groups to facilitate clinical diagnosis and treatment. Group I includes patients who exhibited anterior ulnar plastic deformity concurrent with a radial neck fracture and anterior radiocapitellar joint dislocation. Group II encompasses instances of an ulnar fracture in conjunction with a posterior radial neck fracture and posterior radiocapitellar joint dislocation. Group III, the most prevalent subtype, involves an ulnar shaft fracture associated with a radial neck fracture.

## 6 Pathophysiological Characteristics of Chronic Monteggia Fracture

### 6.1 Osseous Structural Alterations

In numerous patients with chronic Monteggia fractures, malunion or nonreduction of the ulnar fracture frequently results in an ulnar bow, presenting either anteriorly or laterally angulated, and ulnar shortening or angular deformity further disrupts the radioulnar joint congruity, which is a key factor impeding the reduction of the radial head, a critical factor [38, 39, 40]. Concurrently, prolonged dislocation can induce hypertrophy and deformation of the radial head, radial neck thinning, and trochlear dysplasia, even leading to deformities of the capitellum [38], which significantly compromises joint function and stability. Owing to altered upper limb biomechanics, chronic radial head dislocation may result in deformities such as cubitus valgus deformity [38, 41].

### 6.2 Soft Tissue and Ligamentous Pathology

In acute Monteggia fractures, the annular ligament may be completely ruptured or partially torn. Research indicates that in chronic Monteggia fractures, specifically Bado type I and Bado type III fractures, tears are frequently observed on the joint capsule at the lower margin of the ligament, whereas the annular ligament often remains intact. However, even when radiographic imaging confirms radial head reduction, the annular ligament may become entrapped within the radiocapitellar joint, thereby affecting joint stability [42]. Furthermore, chronic radial head dislocation can lead to contracture and fibrosis of the surrounding soft tissues, forming dense scar tissue around the radial head, which complicates reduction [43].

### 6.3 Neurological Dysfunction

Studies suggest that in Bado type I patients, the ulnar nerve may be elongated and compressed due to the combined effects of cubitus valgus deformity, the formation of osteophytes within the cubital tunnel, and subsequent canal stenosis, potentially leading to ulnar nerve palsy [41]. Patients may present with sensorimotor deficits in ulnar nerve distribution. In Bado type III Monteggia fractures, radial head dislocation may compress the posterior interosseous nerve (PIN) posteriorly at the radiocapitellar joint, resulting in corresponding neurological dysfunction [44]. Prolonged radial head dislocation may also cause fibrosis of the compressed nerve, further impacting nerve function [44]. Moreover, research indicates that local soft tissue injuries and hematoma formation can also compress nerves, leading to associated neurological dysfunction [44].

### 6.4 Joint Degeneration and Functional Impairment

Chronic radial head dislocation may lead to joint degeneration and functional impairment, manifesting as pain during activity and limited joint motion, including extension, flexion, pronation, and supination, along with elbow stiffness and weakness. Secondary osteoarthritis may develop due to articular cartilage wear [38, 45]. Cubitus valgus deformity and valgus instability may further exacerbate joint degeneration [38, 41].

## 7 Diagnosis

### 7.1 Clinical Presentation

#### 7.1.1 Elbow Deformity and Abnormal Appearance

Elbow deformity and abnormal appearance are characteristic features of chronic Monteggia fractures [14]. Owing to unreduced radial head dislocation, patients may present with a visible or palpable dislocated radial head forming an abnormal prominence on the lateral aspect of the elbow, and some patients with chronic Monteggia fractures may even exhibit cubitus valgus deformity [14, 46, 47]. If the ulnar fracture is not anatomically reduced correctly, nonunion or angular deformity may occur, further affecting radial head stability and exacerbating elbow deformity [6, 14, 48].

#### 7.1.2 Limited Motion and Functional Impairment

Patients with chronic Monteggia fractures often exhibit limited elbow flexion, extension, pronation, and supination. Limited elbow flexion and extension manifests as a reduced range of elbow flexion and extension, with complete extension being particularly difficult [43, 49]. Rotational dysfunction of the elbow presents as significantly limited forearm pronation and supination, significantly affecting daily activities such as turning doorknobs and writing [8, 49]. If chronic Monteggia fractures are not effectively treated for a long time, soft tissue contracture and fibrosis may lead to elbow stiffness, further exacerbating the loss of joint mobility [8, 50].

#### 7.1.3 Pain and Discomfort

Unlike acute Monteggia fractures, fractures in chronic cases essentially heal, and pain may be related to joint instability, cartilage degeneration, or secondary osteoarthritis [8, 14].

#### 7.1.4 Neurological Symptoms

Compression of the ulnar nerve and the PIN in chronic Monteggia fractures may result in motor and sensory dysfunction in the areas they innervate, resulting in muscle weakness, numbness, and reduced sensory function [7, 8, 41, 51].

### 7.2 Imaging Modalities

#### 7.2.1 X‐Ray

As a fundamental screening tool, anteroposterior and lateral radiographs of the elbow joint and forearm provide an initial assessment of radial head dislocation, ulnar malunion, and degenerative changes in the humeroradial joint [1, 2, 7, 14, 43, 52]. However, studies have indicated a high rate of missed diagnoses with conventional radiography, particularly in cases of radial head dislocation, which is prone to being overlooked, especially when the child's skeleton is not fully ossified [18, 53].

**FIGURE 4:**
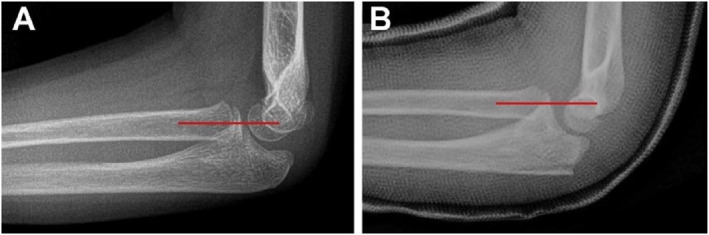
Example of a radiocoronoid line. (A) Lateral radiograph of the uninjured elbow demonstrating the radiocapitellar line intersecting the capitellum. (B) Lateral radiograph of a Monteggia lesion, with the radiocapitellar line anterior to the capitellum. Reproduced from Ref. 7 with permission from ELSEVIER, copyright 2020.

Currently, the most commonly used indicator in clinical practice for evaluating radial head dislocation is the RCL, proposed by Storen [54]. Under typical circumstances, the RCL, which is determined by a line drawn through the center of the radial neck and head, should intersect the capitellum on a lateral radiograph, regardless of the elbow's flexion or extension, and a deviation in the RCL suggests radial head dislocation [7, 54] (Figure 4). With further research, the limitations of the RCL have been gradually recognized. Fader [55] and Tan et al. [56] suggested that patient age and skeletal development may affect the accuracy of the RCL and that the accuracy of the RCL is low in the coronal plane. Kunkel [57] et al. reported that the forearm's pronation and supination during radiography and the subjective factors of the examiner may further reduce the reliability of the RCL. To assess radial head dislocation more accurately in the coronal plane, Souder et al. [58] proposed the lateral humeral line (LHL) in 2017. In anteroposterior projection, the LHL is observed along the lateral border of the humeral lateral condyle, running parallel to the distal humeral shaft axis. Normally, the LHL should be aligned parallel to the radial neck cortex [14, 58].

**FIGURE 5:**
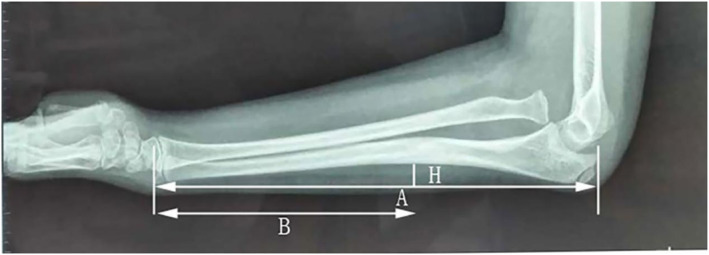
Example of the ulnar bow sign. A dorsal line extends from the olecranon to the distal edge of the ulna. The maximum ulnar bow (MUB), designated MUB (H), is determined by measuring the greatest vertical distance from the straight‐line projection of the ulnar shaft to its dorsal border. Parameter A represents the distance from the olecranon to the distal ulnar epiphysis, and B signifies the distance from the point of the maximum ulnar bow to the distal ulnar epiphysis. The maximum bow ratio is calculated via the formula R‐MUB (H/A), and the location of the maximum bow is recorded as P‐MUB (B/A). Reproduced from Ref. 60 with permission from Nature Publishing Group, copyright 2022.

Ulnar shortening and angular deformities are common malunions in chronic Monteggia fractures. The “ulnar bow sign,” proposed by Lincoln et al. [59] in 1994, is a commonly used evaluation indicator in clinical practice. From a lateral view, a dorsal line is defined as extending from the olecranon to the distal edge of the ulna, and ulnar shaft points exceeding a 1 mm deviation from this line suggest significant ulnar curvature [14, 59]. The maximum ulnar bow (MUB), representing the greatest vertical distance from the dorsal ulnar border to a straight line, was normalized to the ulnar length to derive the MUB ratio (R‐MUB), accounting for radiographic magnification variations [60]. The position of the MUB was quantified as the ratio of the distance from the apex of the bow to the distal ulna to the ulnar length (P‐MUB), with a closer P‐MUB to the middle of the ulna or a larger R‐MUB correlating with a greater osteotomy angle (Figure 5) [60]. The limitation of the ulnar bow sign lies in its high reliance on standard lateral radiographs of the elbow joint [61, 62]. However, obtaining standard lateral radiographs is difficult because of factors such as poor cooperation, leading to an inability to correctly assess the ulnar curvature. Additionally, the anatomical differences in the ulna of children increase the difficulty of applying the ulnar bow sign.

Chronic radial head dislocation may also result in radial head hypertrophy, which can be assessed by measuring the head‒neck ratio on lateral radiographs. In lateral radiographs, the head‒neck ratio was calculated by dividing the maximum metaphyseal width adjacent to the growth plate by the minimum neck width proximal to the bicipital tuberosity. A head‒neck ratio greater than 1.5 is indicative of radial head hypertrophy [38, 63]. Some patients with chronic Monteggia fractures may develop osteoarthritis due to a lack of effective treatment, which is characterized by joint space narrowing and osteophyte formation on radiographs [64, 65].

#### 7.2.2 Three‐Dimensional CT Reconstruction

Three‐dimensional CT can clearly assess ulnar angulation, the degree of radial head dislocation, and the congruity of the joint surface [14, 66], as well as evaluate the presence of intra‐articular bony fragments [49]. This is crucial for developing treatment plans for complex deformities such as combined radial neck fractures or nonunion [67].

#### 7.2.3 MRI Examination

MRI is highly effective in identifying soft tissue pathologies, such as annular ligament entrapment or fibrosis, capsular contracture, and tendon adhesions while also enabling precise assessment of radial head size and joint surface integrity [47, 68, 69]. Furthermore, MRI plays a crucial role in diagnosing Monteggia fracture equivalents, providing valuable information to guide surgical decision‐making [66].

#### 7.2.4 Ultrasound Examination

Ultrasound examination is noninvasive, does not involve ionizing radiation, and allows for dynamic imaging. It can assess almost all major nerves in the limbs, especially for detecting nerve injuries that are unable to be identified with traditional X‐rays, making it a powerful tool for diagnosing and evaluating traumatic neuropathy [70]. Ultrasound technology helps to assess lesions of the superficial radial nerve (SRN) and the PIN preoperatively in children with chronic Monteggia fractures [70]. It can also be used to assess radial head dislocation and annular ligament entrapment, although its accuracy has not been fully established [69].

#### 7.2.5 Arthrography

Elbow arthrography can enhance the detection of fracture sites and radial head dislocations, which may be challenging to assess via conventional radiography, particularly in young children. Furthermore, arthrography can assist in evaluating the reduction of the radial head during surgery, thereby informing the selection of the surgical approach [28, 71].

### 7.3 Differential Diagnosis

Congenital Radial Head Dislocation (CRHD) is relatively rare and typically results from congenital malformations of the radial head; it presents without a clear history of trauma and is often incidentally discovered through imaging studies [72]. Clinical manifestations are often subtle, with patients initially being asymptomatic or experiencing only mild limitations in joint movement. As a patient ages, symptoms such as pain, deformity, and restricted elbow function may gradually develop. Radiographic findings include radial head dislocation with capitellum absence or deformity, a dome‐shaped radial head, and relative shortening of the ulna or overlength of the radius [72, 73, 74].

Proximal radioulnar synostosis can arise from congenital fusion of the radius and ulna or from heterotopic ossification following forearm trauma [75, 76]. Patients typically present with pain and partial or complete restriction of forearm rotation, and radiographic findings demonstrate varying degrees of bony fusion [75, 76].

Traumatic isolated radial head dislocation is a relatively rare condition that is typically associated with a clear history of trauma. In the acute phase, patients exhibit pain, swelling, and limited movement in the affected elbow. If left untreated, it can progress to chronic radial head dislocation, leading to persistent elbow pain, stiffness, and restricted motion. Radiographic findings include isolated radial head dislocation, a negative radioulnar line sign, and the absence of fractures in the ulna or radius [77].

## 8 Treatment

The primary therapeutic objective in the management of chronic Monteggia fractures in pediatric patients is to achieve anatomical reduction, concurrently restoring the anatomical position of the radial head and the ulnar alignment, thereby reestablishing joint stability [7, 14, 78, 79]. Surgical intervention is typically required for chronic Monteggia fractures in children due to the frequent occurrence of ulnar malunion, persistent radial head dislocation, and even cubitus valgus, which are anatomical alterations that cannot be corrected through conservative treatment. Furthermore, surgical management is the principal approach for preventing and addressing complications such as neurological dysfunction, limited elbow joint mobility, and osteoarthritis in patients with chronic Monteggia fractures [4, 5, 8, 43, 45, 52, 80]. In the management of chronic Monteggia fractures, both watchful waiting and conservative treatment strategies have fallen out of favor, with limited discussion in recent literatures [43, 47, 81]. Current consensus advocates for surgical intervention, even in asymptomatic or mildly symptomatic patients, to reduce the risk of subsequent complications [47]. However, most investigators agree that surgical reconstruction of chronic Monteggia lesions is best suited for patients with minimal dysplastic changes in the radial head or capitellum. MRI evaluation may be necessary to assess dysplasia when planning operative treatment for chronic Monteggia fractures. Current clinical strategies for treating chronic Monteggia fractures in children focus on ulnar osteotomy and radial head reduction, with individualized approaches to ligament reconstruction and fixation methods, although a standardized treatment protocol or guideline remains elusive [4, 8, 10, 11, 12, 40, 43, 45, 48, 49, 52, 82].

### 8.1 Radial Head Reduction

Currently, methods for radial head reduction include both closed and open reduction techniques [40, 64]. Closed reduction includes acute reduction and gradual reduction. Acute reduction involves achieving radial head reduction and fixation through intraoperative ulnar osteotomy to correct ulnar morphology. When acute reduction is unfeasible, a monolateral external fixator (MEF) is employed to gradually adjust the ulnar length and angulation, progressively tractioning the radial head into reduction, a method termed gradual reduction [64]. Research has indicated that, compared with acute reduction, gradual reduction has a greater success rate for radial head reduction [64]. For patients in whom closed reduction fails due to soft tissue contracture or other factors, open reduction of the radial head is often employed. This involves a lateral approach to the elbow joint, incising the joint capsule to expose the radial head, removing any fibrous tissue impeding reduction, and subsequently reducing the radial head [40, 45, 64, 83, 84].

### 8.2 Ulnar Osteotomy

Ulnar malunion is a significant impediment to radial head reduction; therefore, ulnar osteotomy is a critical step in achieving radial head reduction and improving elbow joint function [14]. Commonly employed osteotomy strategies include anatomical osteotomy and overcorrection osteotomy. Anatomical osteotomy facilitates radial head reduction by restoring the normal physiological curvature and anatomical length of the ulna, whereas overcorrection osteotomy involves dorsal angulation of the ulna to greater than 10° to reduce the risk of redislocation [85]. Overcorrection osteotomy may be necessary if stable radial head reduction cannot be achieved, although this may lead to posterior radial head dislocation postoperatively [14, 85]. Musikachart et al. [85] demonstrated that anatomical osteotomy resulted in significantly superior radial head reduction compared with overcorrection osteotomy, with only a subset of patients exhibiting mild radial head subluxation, and long‐term follow‐up outcomes were also superior to those achieved with overcorrection osteotomy.

Research indicates that proximal ulnar osteotomy is more conducive to radial head reduction. Furthermore, the 1/3 to 1/5 region is rich in cancellous bone, promoting rapid bone healing, and the incidence of complications such as nonunion or delayed union is significantly lower than that of mid‐distal ulnar osteotomies [47, 86]. For patients with significant angular deformities in the mid‐distal ulna, osteotomy at this site may be considered, with careful attention given to preserving the integrity of the interosseous membrane (IOM) [87]. In cases of significant ulnar malunion, osteotomy based on the center of rotation angulation (CORA) can restore the normal width of the IOM, correct angular and length deformities of the ulna, and promote stable radial head reduction [88, 89].

### 8.3 Selection of Postoperative Fixation Methods

The choice of postoperative fixation method necessitates a personalized approach, considering factors such as the type of osteotomy and the patient's age. Common fixation methods include external fixation and internal fixation. The external fixation options include the monolateral external fixator (MEF), the Ilizarov mini‐external fixator, and the double‐socket external fixator. The MEF offers minimally invasive characteristics, flexibility, adjustability, and ease of removal, making them suitable for gradual reduction of the radial head [49, 64, 82]. Compared with plate fixation, the Ilizarov mini‐external fixator is minimally invasive and provides a more stable mechanical environment through rods, hinges, and struts, enabling controlled radial head reduction and correction of complex deformities such as ulnar rotational deformities [90]. The double‐socket external fixator allows for optimal reduction of the radial head while stabilizing the ulnar osteotomy site and can reduce the incidence of postoperative radial head subluxation and redislocation through timely adjustments [91]. Common internal fixation methods include plate‐screw fixation and Kirschner wire (K‐wire) fixation. Compared with external fixation, internal fixation reduces the risk of pin‐tract infection and facilitates postoperative patient activity, but internal fixation methods such as plate‐screw fixation are more invasive, potentially disrupting the periosteal blood supply, and the removal process is complex, often requiring general anesthesia [45, 90]. K‐wire fixation of the humeroradial joint can further increase the stability of radial head reduction postoperatively [52]. Chen et al. [92] conducted a retrospective study of 249 patients with chronic Monteggia fractures treated with K‐wire fixation of the humeroradial joint between 2013 and 2021, revealing a mean fixation duration of 6.6 weeks, favorable postoperative elbow function scores, and a low risk of complications such as K‐wire breakage, at only 0.8%. However, fixation of the radiocapitellar joint remains a subject of debate. Dai et al. [50], in a retrospective review of 62 pediatric patients with chronic Monteggia fractures from 2005 to 2017, reported that fixation of the radiocapitellar joint did not appear to prevent redislocation. Hybrid fixation methods, which combine limited internal fixation with external fixation, ensure reduction stability and meet the requirements for early postoperative functional exercises [49]. In cases of chronic Monteggia fracture, following radial head reduction and ulnar osteotomy, we posit that the position and stability of ulnar fixation are of paramount importance. Consequently, we do not advocate for routine articular locking of the radiocapitellar joint.

### 8.4 Annular Ligament Reduction or Reconstruction

The necessity of annular ligament reduction or reconstruction in pediatric chronic Monteggia fractures remains a subject of debate. Some researchers contend that correcting the biomechanical alignment solely through ulnar osteotomy is insufficient to maintain the stability of radial head reduction, suggesting that annular ligament reconstruction can minimize postoperative redislocation [12, 17, 49, 51, 93, 94, 95, 96]. Conversely, other scholars suggest that the annular ligament remains intact in patients with Bado Type I and Bado Type III chronic Monteggia fractures; thus, reduction without reconstruction is feasible [11, 42, 97]. Further studies have indicated no significant difference in radial head redislocation rates between the annular ligament reconstruction group and the nonreconstruction group [45, 50, 98]. Song et al. [99] proposed that annular ligament reconstruction is not a mandatory surgical step and should be individualized on the basis of the stability of the radial head.

### 8.5 Radial Head Resection

The current consensus within the scientific community emphasizes the critical role of the radial head as a stabilizer of the elbow joint and forearm, which participates in load transmission, and that radial head resection should be avoided whenever possible. This procedure should be considered a salvage option for chronic Monteggia fractures only when the radial head is irreducible, severely deformed, complicated by advanced osteoarthritis and when radial head resection is contraindicated in cases of complex elbow instability [1, 100, 101, 102]. Research indicates that radial head resection may lead to severe complications, including degenerative changes in the elbow joint and periarticular ossification [1, 101, 102]. Furthermore, owing to incomplete skeletal development in young children, radial head resection may damage the physis, leading to abnormal bone growth. Therefore, radial head resection is not recommended for pediatric patients and is only advised shortly before or after skeletal maturity [101].

## 9 Prognostic Factors

Research indicates that the timeliness of treatment is a critical factor influencing the outcomes of pediatric chronic Monteggia fractures. Each month of delay in treatment within the first year post‐injury increases the risk of radial head redislocation by 1.37 times, emphasizing that early diagnosis and intervention lead to more favorable outcomes [43, 50]. Patient age also influences prognosis; Stragier et al. reported that patients under 6 years of age exhibited good postoperative outcomes, whereas 50% of patients aged 6 years and older developed complications such as subluxation or osteoarthritis [80]. We posit that the severity of the injury and the choice of surgical technique also impact the prognosis.

## 10 Conclusion

The high incidence of missed diagnoses and the challenging treatment of chronic Monteggia fractures in children represent a complex injury pattern within pediatric orthopedics. Currently, a universally accepted classification system for chronic Monteggia fractures in children is lacking, and existing classifications fail to encompass the full spectrum of potential injury types. The establishment of a unified and comprehensive classification system could reduce the incidence of missed diagnoses and improve clinical decision‐making. Regarding treatment, standardized protocols and guidelines are currently unavailable. Significant debate persists regarding the selection of surgical techniques and the necessity of annular ligament reconstruction. Furthermore, clinical uncertainties remain, such as whether surgical intervention is warranted for older children with long‐standing radial head dislocation but satisfactory elbow function. The advancement of treatment strategies for chronic Monteggia fractures in children will likely be facilitated by prospective, multicenter studies with large sample sizes and the implementation of comprehensive long‐term follow‐up systems. Moreover, the integration of artificial intelligence in diagnostic processes, the adoption of multidisciplinary consultations, the application of minimally invasive techniques, and the utilization of novel biomaterials may represent promising avenues for future advancements.

## Author Contributions

**Gengze Li:** investigation, formal analysis, writing – original draft, data curation, visualization, project administration. **Yuan Zhang:** conceptualization, supervision, funding acquisition, writing – review and editing. All the authors listed meet the authorship criteria according to the latest guidelines of the International Committee of Medical Journal Editors. All the authors are in agreement with the manuscript for pulication.

## Funding

Youth Program of the National Natural Science Foundation of China (Grant 82303800) and Youth Project of National Clinical Research Center for Child Health and Disorders (Grant NCRCCHD‐2021‐YP‐05).

## Ethics Statement

The authors have nothing to report.

## Consent

The authors have nothing to report.

## Conflicts of Interest

The authors declare no conflicts of interest.

## Permission to Reproduce Material From Other Sources

Figure 1 reproduced from Ref. 31 with permission from ELSEVIER, copyright 2014. Figure 2 reproduced from Ref. 31 with permission from ELSEVIER, copyright 2014. Figure 3 reproduced from Ref. 7 with permission from ELSEVIER, copyright 2020. Figure 4 reproduced from Ref. 7 with permission from ELSEVIER, copyright 2020. Figure 5 reproduced from Ref. 60 with permission from Nature Publishing Group, copyright 2022.

## Data Availability

Data sharing is not applicable to this article as no new data were created or analyzed in this study.
